# Associated factors of periodontitis and predicted study among young man in China: a population-based cross-sectional study

**DOI:** 10.1186/s12889-024-18732-2

**Published:** 2024-05-04

**Authors:** Xiaohui Wen, Hui Li, Shiting Li, Bei Chang, Shichao Chen, Hongcai Li, Caixia Liu, Guangwen Li

**Affiliations:** 1 Department of Epidemiology, The Fourth Military Medical University, Xi’an, 710032 China; 2 School of Public Health, Southwest Medical University, Luzhou, 646000 China; 3 Department of Oral Implantology, The Affiliated Stomatological Hospital, Southwest Medical University, Luzhou, 646000 China; 4 Institute of Stomatology, Luzhou Key Laboratory of Oral & Maxillofacial Reconstruction and Regeneration, Southwest Medical University, Luzhou, 646000 China; 5 Department of Stomatology, The PLA Rocket Force Characteristic Medical Center, Beijing, 100000 China; 6 Department of Stomatology, Shuguang Hospital, Shanghai University of Traditional Chinese Medicine, Shanghai, 201203 China; 7 Xichang People’s Hospital, Xichang, 615000 China; 8 State Key Laboratory of Oral & Maxillofacial Reconstruction and Regeneration, National Clinical Research Center for Oral Diseases, Shaanxi Key Laboratory of Stomatology, Department of Prosthodontics, School of Stomatology, The Fourth Military Medical University, Xi’an, 710032 China

**Keywords:** Hazard ratio, Oral health-related behaviors, Periodontitis

## Abstract

**Background:**

Periodontitis represents the foremost oral condition in young men, strongly correlated with socioeconomic elements and oral health behaviors. This research aimed to assess the prevalence of periodontitis and associated associations with socio-demographics and oral health practices for subsequent Hazard Ratio (HR) estimation.

**Methods:**

A total of 46,476 young men were recruited to the study between August 2022 and October 2023. A questionnaire on socio-demographic factors and oral health-related behaviors related to periodontitis was completed. The standard procedure was used for oral examination. Logistic regression and hazard ratios were used to estimate the influencing factors, whereas the nomogram was used to predict the risk of periodontitis in young men.

**Results:**

A total of 46,476 young men were surveyed and completed the questionnaire. The overall prevalence of periodontitis among young men was 1.74%. Out of these, 1.7% had mild periodontitis and 0.6% had moderate periodontitis. Age and dental calculus were important factors in the periodontal health of young men. This nomogram, which includes 7 easily obtainable clinical characteristics routinely collected during periodontitis risk assessment, provides clinicians with a user-friendly tool to assess the risk of periodontal disease in young men.

**Conclusions:**

Regular dental prophylaxis is crucial for young men to maintain their gingival health and prevent the onset of periodontitis. Dental calculus plays a prominent role in this matter, as it serves as a significant contributing factor.

## Introduction

The group of periodontal diseases primarily encompasses gingivitis and periodontitis, which are both inflammatory conditions. Periodontitis is a chronic and persistent bacterial inflammation that is triggered by a combination of genetic, social, behavioral, and environmental factors [1]. Gingivitis can be identified by its typical symptoms: inflammation of the gums leading to redness, swelling, and bleeding, without any significant periodontal attachment loss (AL) [2]. Most individuals with early mild gingivitis typically experience no pain and seldom encounter spontaneous bleeding. These patients are unaware of their condition or incapable of recognizing it, so no active intervention is necessary [3]. It is highly important to promptly detect and treat untreated gingivitis as it can escalate the risk of periodontitis, a chronic infection of the periodontal supporting tissue. This could further exacerbate future dental and general health problems, and severe periodontitis could seriously compromise the health and functionality of periodontal tissue, being closely related to many systemic diseases [4]. Thus, it is critical to address this issue promptly to maintain overall well-being [5]. To enhance the oral hygiene of the population and provide preventive measures for oral diseases, it is critical to systemically review and educate individuals on proper oral hygiene practices and strategies. Research indicates that the most effective way to manage and prevent periodontitis is by implementing long-term preventive strategies that can prevent the onset of attachment loss, making early detection, diagnosis, and treatment vital to improving the prognosis of periodontitis [3, 5, 6]. The health of the periodontal tissue is highly susceptible to a multitude of factors, including oral hygiene practices and socio-economic psychology. The process of monitoring oral health is crucial for early detection of periodontal diseases, making it essential to implement primary prevention of these diseases by paying attention to the factors that influence periodontal health and the state of oral health. This strategy involves treating potential diseases before they manifest.

Our objective is to enhance the monitoring of oral health status and the identification of health-related factors affecting oral health. We are aiming to increase the understanding of the socioeconomic factors and behaviors that influence oral health in order to assist health care professionals in enhancing the oral health status of young men. By conducting this study, we hope to contribute to the overall improvement of oral health monitoring and the development of effective strategies to promote oral health in this specific demographic.

## Methods

### Study population

From August 2022 to October 2023, all residents across the nation participated in regular oral examinations. Those who did not agree to participate in the study during their physical examination were not included. In line with the World Health Organization (WHO) classification [7], we considered individuals under 45 as young adults. Overall, 46,476 young men were sampled from 31 provinces nationwide, as detailed in Table 1. The research design involved a cross-sectional study, where the participants were asked to complete a questionnaire about their socio-demographic characteristics and oral health behaviors. The questionnaire included questions on age, gender, ethnicity, educational level, marital status, household income, tobacco and alcohol consumption, tooth brushing habits, and oral health status. The study also included clinical examinations of the participants’ periodontal health status.

**Table 1:** The characteristic of periodontitis among young man

Variable	Category	*N*	Mild periodontitis		Moderate periodontitis	
			*N*	%	*N*	%
Education level	High school or below	20,662	366	1.7	128	0.6
	Junior college degree	20,413	276	1.3	37	0.1
	Undergraduate degree or above	5595	69	1.2	36	0.6
Ethnic group	Han nationality	36,688	528	1.4	163	0.4
	Zhuang nationality	989	49	4.9	3	0.3
	Hui nationality	8974	131	1.4	35	0.3
	Others	19	3	15.7	0	0
Area	City	8665	123	1.4	29	0.3
	Town	10,879	137	1.2	32	0.2
	Rural	27,126	451	106	140	0.5
Occupation	Brain work	3245	22	0.6	8	0.2
	Physical work	43,425	689	1.5	193	0.4
Region	Eastern region	13,590	240	1.7	46	0.3
	Central region	15,844	248	1.5	61	0.3
	Western region	17,236	223	1.6	94	0.5
Oral knowledge	Incorrect answer	26,008	405	1.5	109	0.4
	Correct answer	20,662	306	1.4	92	0.4
Tooth brush frequency	< 2 times/day	17,764	281	1.5	72	0.4
	≥ 2 times/day	28,906	430	1.4	129	0.4
Toothpaste	Ordinary toothpaste	16,790	257	1.5	68	0.4
	Medicated toothpaste	9301	140	1.5	40	0.4
	Fluoride toothpaste	12,888	182	1.4	51	0.3
	Others	7691	132	1.7	42	0.5
Calculus	No	24,251	303	1.2	111	0.4
	Yes	22,419	408	1.8	90	0.4

In accordance with the classification criteria established by the National Bureau of Statistics, China’s 31 provinces can be categorized into three major regions: eastern, central, and western. This classification allows for a more thorough understanding of the diverse geographical, economic, and cultural characteristics that define China [8]. The regions of northern, central, and southern China represent the fundamental building blocks of the nation’s economic, political, and cultural landscape. Within these regions, there are certain key features and differences that distinguish them from one another. The eastern region is composed of 11 provinces and is recognized for its economic dynamism and advanced infrastructure. The central region is comprised of 8 provinces, which are characterized by their strong industrial base and historical significance. The western region, encompassing 12 provinces, is renowned for its rich natural resources and vast agricultural areas.

The research was conducted in accordance with the ethical principles of the Fourth Military Medical University’s Ethics Committee for Medical Research, and all participants provided written informed consent before taking part in the study.

### Oral examination

The dental examination was meticulously performed by our highly proficient and competent dental examiners in our modern, state-of-the-art medical offices. A wide assortment of sophisticated instruments was utilized to guarantee a comprehensive oral examination, such as the celebrated metallic CPI (Community Periodontal Index) probe, which provides precise and exact measurements. In addition, overhead mirrors equipped with advanced LED (Light Emitting Diode) lights were deployed to illuminate even the most inaccessible areas, thus ensuring that no aspect is overlooked during the examination process. Our primary consideration was the comfort and convenience of the patients, and we utilized modern, portable dental chairs that provide flexibility and ease of movement.

All dental examiners have gone through a comprehensive training regimen and calibration process supervised by seasoned periodontists, ensuring their proficiency and expertise in assessing periodontal health. Periodontal examination was performed by assessing attachment loss (AL) and periodontal probing depth (PPD) at six sites per tooth without considering third molars. The severity grading of periodontitis was based on the classification system developed by PI Eke [9]:

1. No periodontitis: No evidence of mild, moderate, or severe periodontitis.
2. Mild periodontitis: ≥2 interproximal sites with AL ≥ 3 mm, and ≥ 2 interproximal sites with PD ≥ 4 mm (not on same tooth) or one site with PD ≥ 5 mm.
3. Moderate periodontitis: ≥2 interproximal sites with AL ≥ 4 mm (not on same tooth), or ≥ 2 interproximal sites with PD ≥ 5 mm (not on same tooth).
4. Severe periodontitis: ≥2 interproximal sites with AL ≥ 6 mm (not on same tooth) and ≥ 1 interproximal site with PD ≥ 5 mm.

Throughout the process, our skilled dental experts made careful use of the CPI probe (DE-485, Majestic, UK), delicately inserting it into the gingival sulcus or pocket to conduct an exhaustive exploration of the full extent of the sulcus or pocket. They ensured every movement was gentle and precise, following the natural contours of the tooth root surface.

Upon concluding our clinical examinations, patients diagnosed with periodontitis received comprehensive reports outlining their oral health status. These reports allowed them to fully understand the extent of their condition and provided them with the necessary information to take further steps to address their unique oral health needs. It was recommended that they consult with an oral health specialist for further evaluation and treatment.

### Self-assessment questionnaires of oral health

The step in the comprehensive protocol involved a detailed and standardized procedure for investigator briefings, designed to provide examinees with a detailed explanation of the self-administered questionnaire they would be required to complete. This questionnaire was carefully constructed and based on data collected from the Fourth National Oral Health Survey [10], incorporating a broad range of questions designed to provide a comprehensive and relevant analysis of respondents’ oral health status. The questionnaire encompassed a variety of facets, including the general information of the participants, their oral health routines, indicators of poor oral hygiene, and the degree of oral health awareness within their families. By implementing such a thorough questionnaire, our primary aim was to guarantee a comprehensive understanding of the examinee’s oral health condition, thereby facilitating a comprehensive approach to their dental examination.

A comprehensive review process was undertaken by an experienced auditor, who meticulously scrutinized each answer to detect and rectify any possible errors or omissions. This meticulous review aimed to uphold the utmost reliability and accuracy of the gathered data. For the sake of maintaining the highest standards of data integrity, any responses that were determined to be incomplete or unsatisfactory were discarded. This ensured the results were reliable and valid, providing useful insights to organizations and individuals.

### Statistical analysis

EpiData 3.0 software (The EpiData Association, Odense, Denmark) was utilized to input data, which was reviewed and confirmed by two individuals. The impact of gender, parental education, dental floss use, and daily brushing frequency on results was also taken into account, and these factors were integrated as confounding variables into the statistical analysis [11].

Using the logistic model, we identified potential influencing factors by generating *P* ≤ 0.10 in our analyses. These factors were then considered in the subsequent analysis. To estimate hazard ratios and 95% confidence intervals, we employed restricted cubic spline (RCS). Stratified analysis was conducted to calculate the risk ratios of periodontitis occurrence at various predicted ages. Additionally, the nomogram prediction was confirmed to accurately depict the risk of periodontitis occurrence. All statistical analyses were performed with the software R (version 4.2.2).

### Ethical considerations

The study protocol was reviewed and approved by the Fourth Military Medical University’s Ethics Committee for Medical Research. Following this, written informed consent was obtained from the participants of the research study. Furthermore, the confidentiality and protection of the participant’s personal information during and after the research process was ensured.

## Results

### Basic features and characteristics

In this survey, 46,476 young men were questioned and completed a questionnaire. The overall prevalence of periodontitis among the participants was 1.74%. This figure comprised 1.7% with mild periodontitis and 0.6% with moderate periodontitis.

The study found that the prevalence of periodontitis was highest among those with the lowest education levels, with a prevalence of 10.5% among high school educated or less, 4.3% among technical secondary education, 3.0% among undergraduate degree or higher, and 2.2% among those with higher education levels. The study also found that the prevalence of periodontitis was higher among males than females, with a prevalence of 3.3% for males and 2.8% for females.

In total, 691 cases of periodontitis were observed in Han nationality, accounting for 1.88% of the total. There were 52 cases of periodontitis in Zhuang nationality, accounting for 5.26%. Similarly, Hui nationality had 166 cases of periodontitis, amounting to 1.85%. Finally, other ethnic groups had three cases of periodontitis, contributing to a total of 15.79%.

Considering the geographical location, a total of 152 people with periodontitis resided in the city, representing 1.75% of the total population. 169 individuals with periodontitis were located in the township, accounting for 1.55%. 591 people with periodontitis were found in rural areas, comprising 2.18% of the total population.

In terms of occupation, 30 individuals with periodontitis worked as mental workers, representing 0.92% of the total population with periodontitis. 882 individuals were manual workers, accounting for 20.3%. Geographically, 286 individuals with periodontitis were from the eastern region, which represented 2.10% of the total. 309 individuals were from the central region, accounting for 1.95%. 317 people were from the western region, comprising 1.84%.

Among those with periodontitis, the proportion of people who had correct cognition and habits about oral health was 1.98%, with 514 individuals answering correctly. On the other hand, 1.93% of those with periodontitis had incorrect answers. Concerning oral hygiene habits, 1.99% of people with periodontitis brushed their teeth less than twice a day, 1.93% brushed their teeth more than or equal to twice a day, 1.93% used regular toothpaste, 1.94% used drug toothpaste, 1.81% used fluoride-containing toothpaste, and 2.26% used other types of toothpaste. In terms of oral hygiene outcomes, 1.71% had no calculus, and 2.22% had calculus.

### Association between Basic features and characteristics and periodontitis

Table 2 provides a breakdown of the relationships between socio-demographic, oral health behavior and attitudes, and periodontitis-related oral health status variables. The findings highlight that an increased risk for periodontitis is linked to older age groups and those with lower education levels.

**Table 2:** Association between socio-demographic, knowledge, behavior factors and periodontitis prevalence according to univariate logistic regression analysis

Variables	OR	*P* value
Age (year)	0.0615(0.04,0.08)	< 0.001
Education level		
High school or below	Ref.	
Junior college degree	-0.352(-0.51, -0.20)	< 0.001
Undergraduate degree or above	-0.169(-0.39,0.05)	0.013
Ethnic group		
Han nationality	Ref.	
Zhuang nationality	1.18(0.87,1.47)	< 0.001
Hui nationality	-0.276(-0.49, -0.068)	0.010
Others	2.4(0.92,3.53)	< 0.001
Area		
City	Ref.	
Town	-0.1(-0.33,0.14)	0.402
Rural	0.167(-0.02,0.37)	0.093
Occupation		
Brain work	Ref.	
Physical work	0.889(0.51,1.31)	< 0.001
Region		
Eastern region	Ref.	
Central region	-0.125(-0.30,0.05)	0.153
Western region	-0.206(-0.39, -0.03)	0.019
Oral knowledge		
Incorrect answer	Ref.	
Correct answer	0.08(-0.06,0.22)	0.271
Tooth brush frequency		
< 2 times/day	Ref.	
≥ 2 times/day	-0.08(-0.22,0.07)	0.278
Toothpaste		
Ordinary toothpaste	Ref.	
Medicated toothpaste	0.0563(-0.14,0.25)	0.572
Fluoride toothpaste	-0.0569(-0.24,0.12)	0.538
Others	0.133(-0.07,0.33)	0.197
Calculus		
No	Ref.	
Yes	0.343(0.20,0.49)	< 0.001

Furthermore, the study did not find an association between a high prevalence of periodontitis and poor knowledge and attitudes about oral health, including misconceptions such as “bleeding during brushing is normal” and “brushing does not prevent periodontitis.” However, it was evident that oral health behaviors, such as brushing less than twice daily, can increase the likelihood of developing periodontitis. The presence of calculus, particularly a large amount, was identified as a significant risk factor for periodontitis, and individuals with calculus had a 34.3% higher risk of developing periodontitis than those without calculus.

### Predicted hazard ratios of periodontitis

**Fig. 1:**
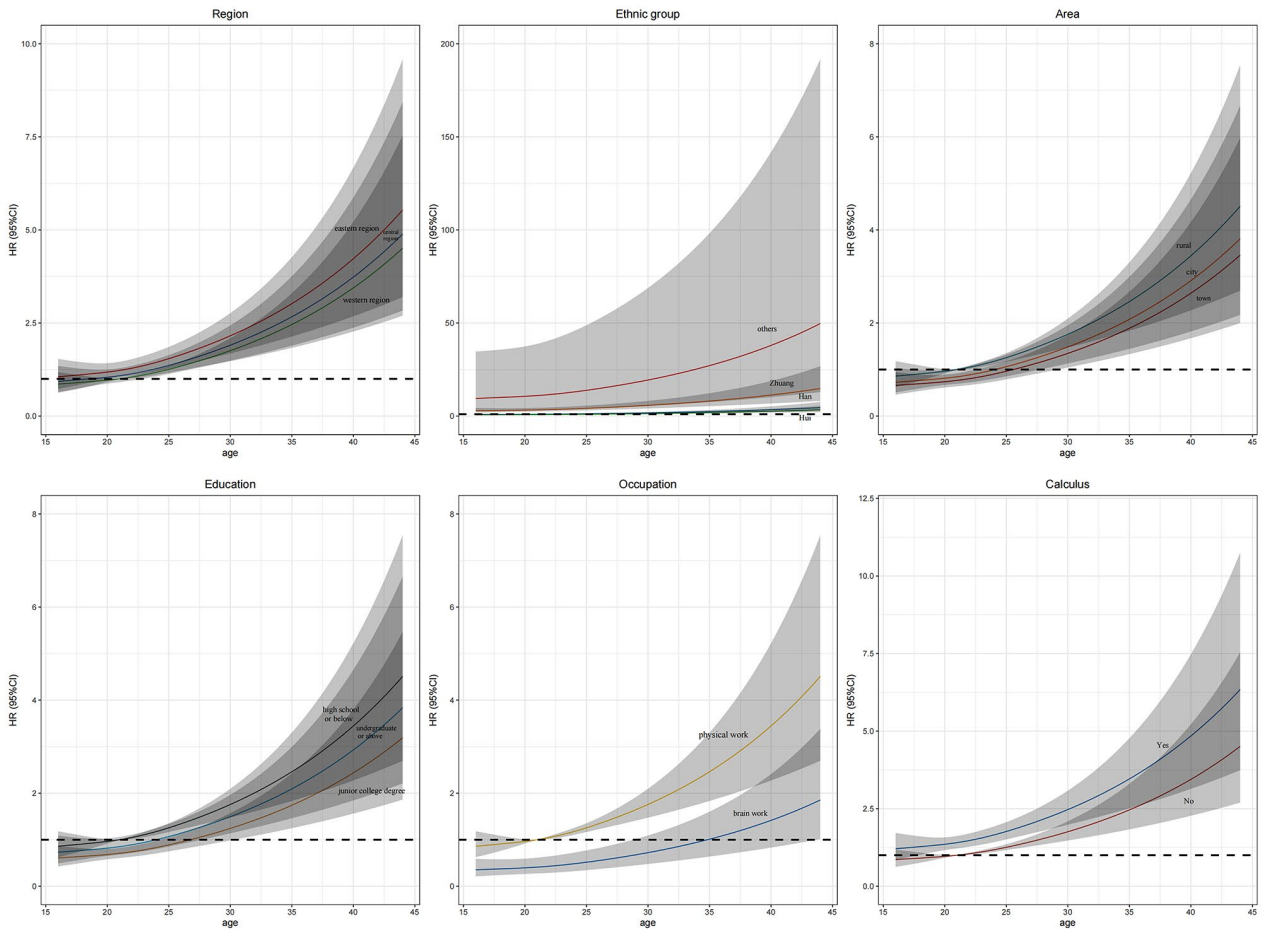
Predicted hazard ratios of periodontitis with age and different predictors in young man. Hazard ratios (HR) are indicated by solid lines and 95% confidence intervals (CI) by shaded areas

The observed relationship between age and periodontitis, illustrated in Fig. 1, is generally consistent across different variables. While age appears to be positively associated with factors such as region, area, education, and occupation, the correlation between age and these variables is relatively weak. However, when the data is disaggregated by ethnic groups and calculus, the association between age and periodontitis becomes more pronounced.

To provide more insight into these relationships, we used linear models to establish the specific connections between age and the factors shown in Fig. 1. The risk ratios associated with periodontitis covered a broad range, ranging from 1 to an incredible 50. These figures indicate the comparative risk of developing periodontitis in relation to age and the specific factor under consideration.

These results indicate that while age is indeed a significant factor contributing to the occurrence of periodontitis across various predictive indicators, the magnitude of this association may vary depending on the factor under examination. This finding underscores the complexity of the age-periodontitis connection, highlighting the necessity for more thorough research to fully comprehend the underlying biological mechanisms and potential therapeutic interventions to mitigate the risk of this oral health issue.

### Nomogram prediction of periodontitis occurrence

**Fig. 2:**
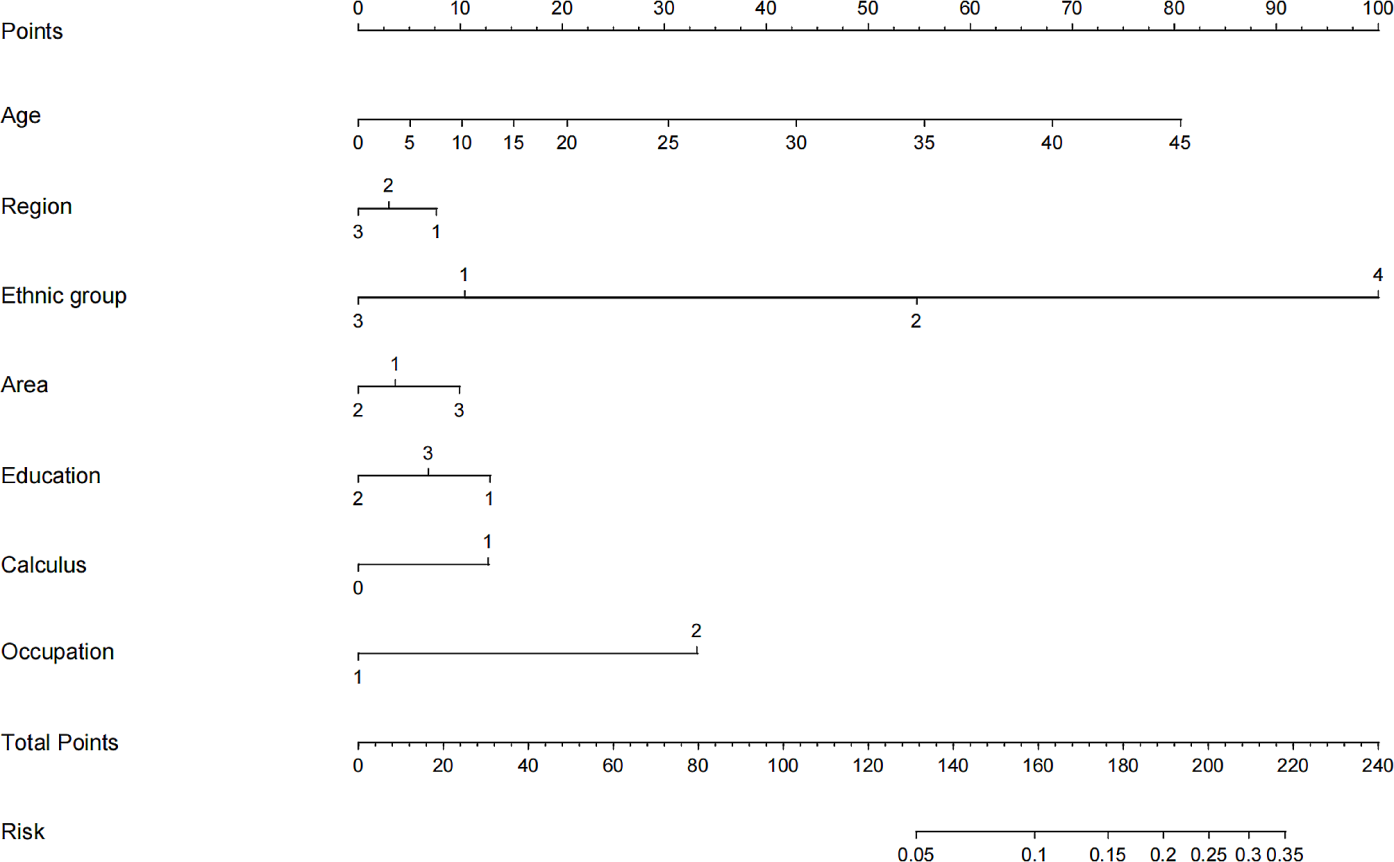
The nomogram was used to predict the risk of periodontitis occurrence among young man. (Draw a line perpendicular from the corresponding axis of each risk factor until it reaches the top line labeled “Points”. Sum up the number of points for all risk factors then draw a line descending from the axis labeled “Total Points”. Region, 1: eastern region, 2: central region, 3: western region; Ethnic group, 1: Han nationality, 2: Zhuang nationality, 3: Hui nationality; 4: Others; Area, 1: city, 2: town, 3: rural; Education, 1: high school or below, 2: junior college degree, 3: undergraduate degree or above; Occupation, 1: brain work, 2: physical work; Calculus, 0: no, 1: yes.)

Binary logistic regression analysis was used to determine whether a binary outcome such as periodontitis would occur. A prediction model was developed using several factors, including age, region, ethnic group, area, education level, occupation, and calculus. The selection of these factors was based on their potential influence on the development of periodontitis. In order to graphically represent the prediction model, a nomogram was created (Fig. 2). A nomogram is a useful tool for estimating probabilities based on the values of different predictors. For this model, the nomogram features a point-scale axis for each predictor, with values for each factor corresponding to a specific score. The total score for a person is calculated by adding the scores for each factor together. For example, if a person is 45 years old, lives in a specific region, belongs to a particular ethnic group, has a specific area of residence, has achieved a particular level of education, had a specific occupation, and had a certain amount of calculus, their total score is determined by adding the scores for each factor.

This prediction model and accompanying nomogram can be used as a practical tool to estimate the probability of periodontitis occurrence based on the values of the seven predictors. It allows health care professionals and researchers to conveniently assess the risk of periodontitis, enabling them to make informed decisions about prevention and treatment strategies. The total score of the model can be mapped onto a lower total score scale on the nomogram, allowing for an estimate of the risk of developing periodontitis. The lower total score scale represents the probability of periodontitis occurrence, ranging from low to high. Thus, simply by locating the total score on the nomogram and dragging it down to the lower total score scale, the corresponding probability can be determined.

## Discussion

Despite numerous epidemiological studies consistently identifying periodontitis as a common disease [12], there is evidence of considerable variation in its prevalence rates across various populations and regions. These disparities may be attributed to inter-population variability, but it should be noted that the differences may also be a result of varying diagnostic criteria used across studies. Periodontitis prevalence ranges from a low of 1.7% to a high of 3.7% in different regions and populations [13–15]. The results of our study suggest that the prevalence of unhealthy periodontal status among young Chinese men aged 15–44 years is low, which agrees with findings from other studies [16].

The CPI appears to be a pragmatic and suitable methodology for large-scale oral health surveys. The CPI was utilized to evaluate gingival health, with a score of 1 used to indicate gingival inflammation. Consequently, the prevalence of periodontitis (or gingival inflammation) was reported to range from 37.4 to 99% among various populations [17, 18]. As demonstrated in previous studies, the CPI may underestimate the prevalence of periodontal conditions when compared to more comprehensive full mouth protocols [19]. The varying interpretation of periodontitis’ definition and various clinical methods for evaluating gingival health make it challenging to compare the findings of various epidemiological studies and accurately gauge the prevalence of periodontitis.

Due to financial and time limitations, we chose the WHO approach to gingival bleeding, which is particularly suited for epidemiological field studies. Our study found an extremely low prevalence of clinical AL ≥ 4 mm in young man, at 0.1%. Given this finding, precision calibration probes are generally unnecessary for detecting PD or AL in field studies. We therefore chose the CPI probe as the measurement tool. A study by Kingman et al. suggests that full-mouth recording excluding wisdom teeth can be considered the gold standard for clinical examinations [20, 21]. In our survey, all teeth except the third molars were probed to assess gingival health. Finally, the estimation of disease prevalence, assessment of related risk factors, and disease surveillance require a definition of disease. However, in the past, there was no precise and generally accepted definition of periodontitis, and the diagnostic criteria for periodontitis were constantly being revised. The joint AAP/EFP (the American Academy of Periodontology and the European Federation of Periodontology) workshop in 2018 reported the definition of periodontitis, in which patients with intact periodontium would be diagnosed as having periodontitis if found with a BOP (Bleeding on Probing) score ≥ 10%. Gingival hemorrhage is a sensitive indicator of gingival inflammation and recording gingival hemorrhage is recommended by the WHO for epidemiological investigations because it is economical and requires minimal/no technology [22]. Therefore, to better monitor the prevalence and severity of periodontitis among young men, we adopted periodontal pocket recordings on all teeth in our study to define and grade periodontitis.

Several factors contribute to the development of periodontitis, including oral health behaviors, gender, oral hygiene practices, dental calculus formation, and socioeconomic status, the principal cause of plaque-induced periodontitis is the presence of bacterial biofilm in the oral cavity [11, 23–25]. It is widely acknowledged that dental calculus, in its calcified form, serves as an ideal breeding ground for bacterial biofilm, which is seen as an important secondary contributor to the development and progression of periodontal diseases [23]. Previous studies have discovered that dental calculus is a significant risk factor for periodontitis, with evidence suggesting its prevalence in patients suffering from the condition is significantly higher than those without [15, 26, 27]. Our research determined that individuals diagnosed with periodontitis and calculus displayed a significantly elevated HR compared to those who were periodontitis-free.

The findings of our study indicate that while age does play a role in the development of periodontitis across various predictors, the magnitude of this association may vary depending on the specific factor under scrutiny. This underscores the complexity of the relationship between age and periodontitis, calling for further research to fully comprehend the underlying mechanisms and potential interventions to mitigate the risk of this oral health condition. Specifically, our study discovered that the HR of other nationalities was as high as 50, which is considerably higher than that of Han nationality, Hui nationality, and Zhuang nationality. The most probable cause for this observation is the relatively smaller population size and increased variability of these nationalities.

In China, rapid social and economic development has spurred impressive advancements in social development and urbanization. Undeniably, these changes have undoubtedly reshaped lifestyle and practices, potentially impacting oral health and associated behaviors [28]. It is intriguing to note that practices such as brushing twice a day, a common indicator of good oral hygiene, do not consistently serve as protective factors against periodontitis. This surprising observation defies our understanding of the previous research [29].

Several studies have found that a combination of socioeconomic factors such as education, family economic level, dental care access, and behavioral factors contribute to the development of periodontitis [20, 29, 30]. Education level and occupation type play a significant role in the development of periodontal disease. Those with higher education levels tend to have a reduced risk of developing periodontal disease, whereas manual workers often experience a higher risk of periodontal disease. Many lower socio-economic families face challenges such as limited income, limited access to dental services, and poor oral hygiene practices. Studies have shown a strong correlation between low socioeconomic status and a heightened risk of periodontal diseases, an increased prevalence of calculus, and poorer oral hygiene habits [31–33]. In China, economic conditions and consumption levels in rural areas are inferior to those in urban and urban areas. Our research revealed significant disparities in the prevalence of periodontitis. It is plausible that rural youth might lack access to quality oral healthcare services, unlike their urban counterparts. Consequently, it is vital to accentuate the significance of oral health policies that prioritize periodontal health in rural communities and enhance oral healthcare services. Periodontal care initiatives should primarily focus on health promotion and education, standard oral care, and preventive measures. These initiatives have the potential to significantly boost oral hygiene. Interestingly, individuals in the eastern region of China exhibit more HR than those in the central and western regions. Compared to the western and central regions, people in the eastern regions have a higher risk of periodontitis, which may be linked to dietary characteristics or food abundance in the eastern regions.

Our nomogram is a helpful tool for clinicians to determine future risk and provide patients with a personalized assessment [34, 35]. Furthermore, the outcome may be applied as a guide for preventative treatment. To fully evaluate the effectiveness of preventive treatment strategies based on the current risk prediction model, additional comparative studies are needed. Such studies could include the utilization of nomograms, which have been used frequently in disease prognoses and primary applications in predicting the likelihood of an event such as the recurrence of early gastric cancer or renal cancer [36, 37]. In our study, we used a nomogram to predict periodontitis, allowing us to better assess the risk of someone developing periodontitis.

The limitations of this study are considerable and should not be overlooked. The exclusion of the female group from our research is a significant issue that left us lacking in demographic data and overarching population insights. In addition, our research model does not account for factors such as medication usage or oral health history, which could have significantly influenced our findings. Third, in our research, we utilized the older definition, but given that the updated definition has been implemented, the obtained results should be treated with a certain degree of caution. Finally, it is crucial to consider the sample population of this study when interpreting the results. The sample was composed of individuals who were receiving physical examinations, and the predictive model has not been fully validated. As a result, the predictive accuracy of the model for populations outside of this group should be carefully evaluated before its application. While these limitations may detract from the overall reliability of our findings, it is important to acknowledge the significant insights we gained regarding the impact of tobacco use on oral health and the potential implications for future prevention efforts.

## Conclusions

Age and dental calculus are key risk factors for young men throughout China in relation to their periodontal health. This nomogram, a simple tool comprising seven easily obtainable clinical characteristics routinely collected during periodontitis risk assessment, allows clinicians to evaluate the potential risk of periodontal disease in this demographic. Regular dental prophylaxis is vital for young men to maintain healthy gums and prevent the onset of periodontitis.

## Data Availability

The datasets generated and/or analysed during the current study are not publicly available because we have not obtained the consent of all parties involved to publicize their data regarding personal privacy issues, but are available from the corresponding author on reasonable request.

## Abbreviations

AL: Attachment loss
CPI: Community Periodontal Index
HR: Hazard Ratio
